# Aptamer-Based Strategies to Boost Immunotherapy in TNBC

**DOI:** 10.3390/cancers15072010

**Published:** 2023-03-28

**Authors:** Lisa Agnello, Annachiara d’Argenio, Roberto Nilo, Monica Fedele, Simona Camorani, Laura Cerchia

**Affiliations:** Institute of Experimental Endocrinology and Oncology “Gaetano Salvatore”, CNR, Via S. Pansini 5, 80131 Naples, Italy; lisa.agnello@ieos.cnr.it (L.A.); annachiara.dargenio@ieos.cnr.it (A.d.); roberto.nilo@ieos.cnr.it (R.N.); mfedele@unina.it (M.F.); s.camorani@ieos.cnr.it (S.C.)

**Keywords:** immune system, immunotherapy, aptamer, TNBC, active cancer targeting

## Abstract

**Simple Summary:**

Triple-negative breast cancer (TNBC) is an aggressive breast cancer subtype with poor outcomes and limited therapeutic options. It is characterized by a more pronounced immunogenicity compared with other breast cancer subtypes, suggesting immunotherapy is a viable strategy. Aptamers are short oligonucleotides that, similar to antibodies, recognize their protein target with high specificity and affinity. However, compared with antibodies, they present several advantages in terms of size, production, modification, and stability, which make them excellent candidates for the development of novel targeted anticancer therapies. Recently, in order to restore an immunoreactive and anticancer tumor microenvironment, effective aptamer-based strategies have been developed. Here, we discuss the most recent approaches aimed at using aptamers to enhance or restore the anticancer immune response in TNBC.

**Abstract:**

The immune system (IS) may play a crucial role in preventing tumor development and progression, leading, over the last years, to the development of effective cancer immunotherapies. Nevertheless, immune evasion, the capability of tumors to circumvent destructive host immunity, remains one of the main obstacles to overcome for maximizing treatment success. In this context, promising strategies aimed at reshaping the tumor immune microenvironment and promoting antitumor immunity are rapidly emerging. Triple-negative breast cancer (TNBC), an aggressive breast cancer subtype with poor outcomes, is highly immunogenic, suggesting immunotherapy is a viable strategy. As evidence of this, already, two immunotherapies have recently become the standard of care for patients with PD-L1 expressing tumors, which, however, represent a low percentage of patients, making more active immunotherapeutic approaches necessary. Aptamers are short, highly structured, single-stranded oligonucleotides that bind to their protein targets at high affinity and specificity. They are used for therapeutic purposes in the same way as monoclonal antibodies; thus, various aptamer-based strategies are being actively explored to stimulate the IS’s response against cancer cells. The aim of this review is to discuss the potential of the recently reported aptamer-based approaches to boost the IS to fight TNBC.

## 1. Introduction

Triple-negative breast cancer (TNBC) is an aggressive breast cancer (BC) subtype that accounts for approximately 10–20% of all diagnosed BCs. It generally affects younger women, is more likely to recur, and is associated with a poorer overall survival rate compared with other BC subtypes [1,2]. The term TNBC refers to a heterogeneous group of tumors having different histological, genomic, and immunological characteristics and response to therapy, which have in common the lack of estrogen receptor, progesterone receptor, and epidermal growth factor receptor 2 (HER2); thus, TNBC patients are not eligible for hormone or anti-HER2 therapy [2,3].

Based on gene expression profiling, Lehman et al. [4] initially classified TNBC into six distinct molecular subtypes, namely basal-like 1 (BL1), basal-like 2 (BL2), immunomodulatory (IM), mesenchymal (M), mesenchymal stem-like (MSL), and luminal androgen receptor (LAR). Subsequently, the IM and MSL subtypes were removed from the initial classification because it was recognized that their transcriptomic features were not derived from tumor cells but from tumor-infiltrating lymphocytes (TILs) and tumor-associated stromal cells, respectively, abundantly present in the TNBC tumor microenvironment (TME) [5]. Another classification for TNBC was suggested by Burstein et al. [6], who confirmed four subtypes reported by Lehman: LAR, M, and two BL. Basal subtypes described by Burstein, on the basis of immune signaling, are divided into basal-like immune-suppressed, which exhibits downregulation of B cell, T cell, and natural killer (NK) cell immune-regulating pathways and cytokine pathways, and basal-like immune-activated, which is characterized by upregulation of genes controlling B cell, T cell, and NK cell functions. Burstein and colleagues demonstrated that immunosuppressed and immunoactivated tumors have the worst and best prognoses, respectively. Both classifications highlight the immune system (IS) signature as an important prognostic factor in TNBC and a potential target in the treatment of some TNBC subtypes.

The limited options of molecular therapies and the highly heterogeneous nature of these tumors make TNBC the most challenging BC to treat. To date, the standard of care for both early and advanced TNBC remains chemotherapy, generally using combined regimens of taxane, anthracycline, cyclophosphamide, cisplatin, and fluorouracil [7,8]. Unfortunately, chemotherapy efficacy is limited by the high toxicity toward normal cells [9]. Moreover, after a brief initial response to treatment, TNBC tends to reappear in a more aggressive and chemoresistant form, showing a significantly high pathological remission rate [10]. Therefore, it is increasingly necessary to identify specific molecular signatures that can be targeted to establish new effective treatments for TNBC. Years of intense research into TNBC led to the identification of the first molecular therapies.

The first clinically validated biomarker for TNBC therapy was the BRCA mutational status. Up to 19% of TNBC patients carry mutations in genes encoding for BRCA1 and BRCA2, proteins involved in DNA double-strand break repair, resulting in sensitivity to poly-ADP-ribose polymerase (PARP) inhibitors [2,11]. In 2018, the Food and Drug Administration (FDA) approved two PARP inhibitors, Olaparib [12,13] and Talazoparib [14], as monotherapy for the treatment of patients with BRCA-mutated tumors. Currently, the efficacy of PARP inhibitors is also being investigated in BRCA wild-type TNBC [15].

TNBC has been shown to be more immunogenic than other BC subtypes due to its high propensity to generate neoantigens that can be recognized as “nonself” by the adaptive IS [16] and the presence of immune cell infiltrates, which are responsible for developing antitumor immune responses [17,18]. In addition, approximately 20% of TNBC expresses anti-programmed cell death-ligand 1 (PD-L1) [19], a surface protein that binds programmed cell death protein 1 (PD-1) receptors on TILs, turning off their activity. In this way, the PD-1/PD-L1 axis induces an immunosuppressive TME responsible for tumor immune evasion. All these features are fundamental prerequisites for benefiting from immunotherapy. Indeed, in 2019, the FDA approved the first immunotherapy with anti-PD-L1 Atezolizumab monoclonal antibody (mAb) to treat PD-L1-positive unresectable, locally advanced, and metastatic TNBC in combination with nab-paclitaxel [20,21]. In the following 2 years, Pembrolizumab, an anti-PD-1 mAb, was also approved by FDA in combination with standard chemotherapy: first, in 2020, for patients with locally recurrent inoperable or metastatic TNBC and later, in 2021, for newly diagnosed and operable early-stage TNBC by adding the antibody to both neoadjuvant and adjuvant chemotherapy [22]. However, the mentioned therapies are approved only for TNBC expressing high levels of PD-L1, making it necessary to find new strategies to expand the applications of immunotherapy for TNBC.

More recently, the FDA approved the first antibody-drug conjugate (ADC) as a second-line treatment for patients with unresectable locally advanced or metastatic TNBC who have received two or more prior systemic therapies, at least one of them for metastatic disease. The name of this ADC is Sacituzumab govitecan, which is composed of a humanized trophoblast cell surface antigen-2 antibody coupled via a linker to the SN-38 payload, the active metabolite of the topoisomerase 1 inhibitor irinotecan [23].

Oligonucleotide aptamers are highly selective compounds emerging as alternative or complement to mAbs for active cancer targeting, and the application of aptamers as innovative therapeutic agents in different human cancers, including TNBC, is increasing rapidly [24]. In this review, we will first discuss the features of aptamers against cancer-related biomarkers as innovative therapeutics. Then, we will focus on the recently emerged aptamer-based strategies that can prevent immune evasion and stimulate anticancer immune responses, discussing their potential to manage TNBC in the next future.

## 2. Aptamers for Targeted Cancer Therapy

**Figure 1:**
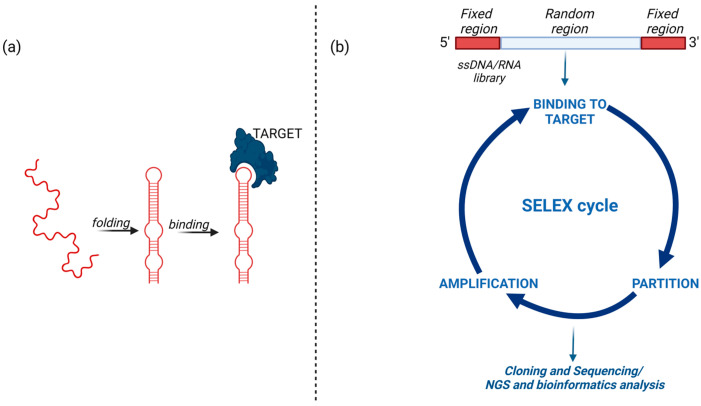
Schematic representation of aptamer binding to its target and key steps of the SELEX method. (**a**) The aptamer adopts a 3D structure to bind to its target; (**b**) The SELEX starts with random libraries of ssDNA or RNA sequences and comprises reiterated rounds of binding to the target, partitioning of the target bound sequences from unbound and amplification of the bound sequences. Finally, the enriched library is analyzed by cloning and sequencing or, in the most recent approaches, high-affinity ligands are identified by next-generation sequencing (NGS) and bioinformatics. Created with BioRender.com (accessed on 2 March 2023).

Aptamers are synthetic, short, single-stranded DNA or RNA oligonucleotides that fold into unique three-dimensional (3D) structures interacting at high affinity and specificity with targets of different natures. Aptamers were first discovered in 1990 when two papers [25,26] described the in vitro SELEX (systematic evolution of ligands by exponential enrichment) technology for aptamer’s generation and introduced the term aptamer, which derives from the Latin word “aptus” (to fit) and the ancient Greek word “meros” (part), thus meaning “fitting parts” (Figure 1).

The in vitro selection starts with the generation of a combinatorial library of oligonucleotides, each containing a central random sequence, approximately 20–100 nucleotides in length, flanked by two fixed regions necessary for primers annealing during amplification and in vitro transcription (to select RNA aptamers). The library is incubated with a target molecule, and a partitioning step is performed to separate oligonucleotides bound to the target from unbound nonspecific sequences. Targets for SELEX can include peptides, proteins, metabolites, carbohydrates, small organic molecules, other structured RNAs, and even whole cells or organisms, and different selection schemes that have been described so far adapted to the nature of the target [27,28,29]. Once eluted from the target, the sequences are directly amplified by PCR in the case of DNA-SELEX or first reverse-transcribed and then amplified in the case of RNA-SELEX. The amplified sequences are used to generate a new oligonucleotide library, which is subjected to a further round of selection. By repeating multiple rounds of incubation, partition, and amplification, this process leads to the generation of sequences with high affinity and specificity for the target (Figure 1).

Aptamers can be considered the nucleic acid version of protein antibodies because they share with them a high binding affinity and specificity for the target (Kd values, 10^−8^–10^−12^ M) and, analogous to antibodies, have a variety of applications as recognition elements, including therapeutics, biosensors, and diagnostics [30].

Aptamers discriminate among highly similar members of the same protein family, protein isoforms, and also proteins that differ in a single amino acid [27]. For example, by a contrast SELEX screening with agarose beads and magnetic beads coupled with wild-type and mutant proteins, respectively, RNA aptamers have been generated able to bind to p53R175, one of the hot spots of p53 mutation, discriminating the mutant protein from wild-type p53 [31].

**Figure 2:**
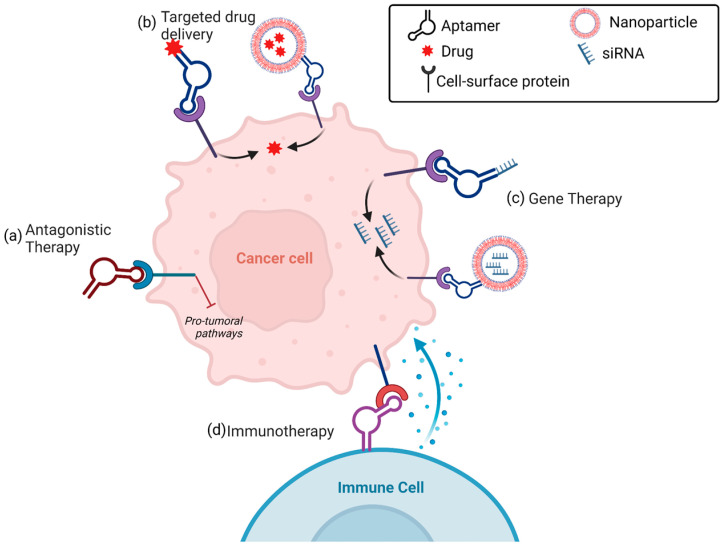
Aptamer-based anticancer therapy. (**a**) Antagonistic therapy: aptamers bind to cancer cell surface targets, inhibiting protumoral pathways. (**b**) Targeted drug delivery: aptamers conjugated to drug-loaded nanoparticles or linked to drugs bind to cell surface targets and internalize into cancer cells, resulting in selective intracellular drug delivery. (**c**) Gene therapy: aptamers decorating small-interfering RNA (siRNA)-loaded nanoparticles or conjugated directly to siRNA, bind to cell surface targets and internalize into cancer cells, resulting in selective gene silencing. (**d**) Immunotherapy: aptamers stimulate immune cells against cancer cells (see text for details). Created with BioRender.com (accessed on 2 March 2023).

Aptamers may function as anticancer therapeutics by different modalities (Figure 2).

As it happens for an antibody, the action of the aptamer as a cancer therapeutic basically relies on its ability to bind to a cancer-related protein target and interfere with its correct functioning, thus ultimately inhibiting tumor development and progression [27,32]. Alternatively, similar to mAbs linked with payloads in ADC [33], aptamers against unique cancer biomarkers can be applied as anticancer tools by exploiting them as targeting agents to transport therapeutic molecules specifically to tumor sites [28,34]. In this regard, the capability of a subset of cell-targeting aptamers to actively internalize into target cells via receptor-mediated endocytosis or micropinocytosis [35,36] has fueled an area of intense research aimed at developing smart aptamer-based modalities for targeted TNBC treatment by conjugation aptamers with chemotherapeutics [37], therapeutic RNAs [38], small inhibitors [39] or nanosystems loaded with drugs [40,41], which need to enter into the cell to exert their anticancer function. Furthermore, recent approaches are also attempting to confer to the aptamers the effector functions, typical of the antibody, of activation of the complement system [42]. Moreover, radiolabeling methods applied to antibodies can be easily used for conjugating aptamers with radionuclides, and the resulting conjugates have great potential in innovative theranostic applications [43]. Even if aptamers can be compared with antibodies for their mode of action, there is no doubt that they have several advantages to successfully replace or complement traditional antibodies for active cancer targeting (Table 1) [44,45]. First, they have smaller sizes (5–15 kDa) and more flexible structures compared to classical antibodies that allow them to penetrate into solid tumors more easily and bind to small and hidden targets otherwise inaccessible to antibodies [46]. Importantly, the production of identified aptamers by chemical synthesis allows to avoid batch-to-batch variability and overcome expensive and labor-intensive steps that are required for antibody production.

**Figure 3:**
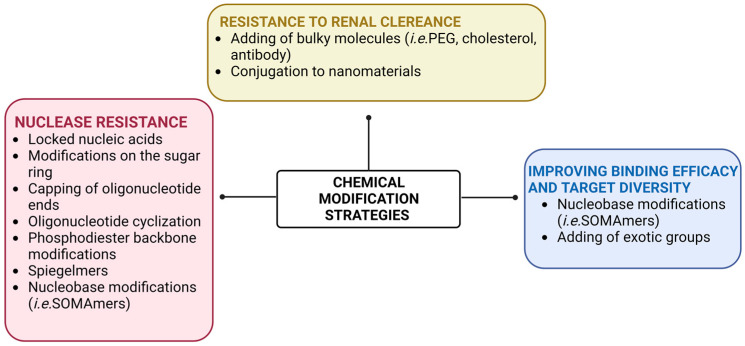
Schematic representation of the most common strategies for chemically modifying aptamers in order to improve their clinical applicability. Created with BioRender.com (accessed on 2 March 2023).

In addition, aptamers tolerate several chemical modifications that improve targeting efficacy, pharmacokinetic profile, and stability in biological environments, which are required for their in vivo applications [47]. As extensively reviewed elsewhere [27,48], the most used modifications applied to aptamers, either during SELEX or post-SELEX, to increase their resistance against nucleases include (Figure 3): the replacement of the 2′-OH groups of ribose with fluoro, methoxy, thiol or amino groups; the capping or the cyclization of the oligonucleotides’ ends; the substitution of the phosphodiester backbone with a phosphorothioate backbone; and the introduction of locked nucleic acids. Moreover, L-aptamers, called spiegelmers, can be generated that are not recognized by nucleases because they are enantiomers of natural nucleic acids. Chemical modifications are also applied to overcome the rapid renal filtration of small-size aptamers by conjugating them to bulky molecules, such as polyethylene glycol (PEG) or cholesterol, thus increasing their circulation time without affecting the accessibility to the target. Sophisticated approaches have also been developed to chemically conjugate aptamers with secondary therapeutics in combination therapy, and interestingly, innovative strategies have been explored to introduce exotic chemical groups in the aptamer molecule to extend their functionality and overcome the lack of chemical diversity in nucleic acids [49]. Another strategy to improve binding affinity, target selectivity, and in vivo bioavailability of aptamers is represented by the generation of slow off-rate modified aptamers (SOMAmers). These are DNA aptamers that bear chemically modified nucleotides functionalized at the 5-position of uridine with moieties that can not only participate in interactions with the target protein but also form novel secondary and tertiary structural motifs that greatly increase the repertoire of targets accessible to aptamers [50].

To date, one extensively chemically modified aptamer (named Macugen), targeting the isoform 165 of vascular endothelial growth factor, has been approved for the treatment of age-related macular degeneration, and eleven aptamers are in clinical trials for the treatment of different human diseases [51,52]. Among them, the anti-nucleolin AS1411 aptamer and the anti-stromal cell-derived factor 1 NOX-A12 aptamer have already completed phase II clinical trials for cancer therapy. Moreover, the anti-protein tyrosine kinase-7 Sgc8 DNA aptamer, labeled with 68 Ga, is in early phase I for assessing its diagnostic value in colorectal patients (ClinicalTrials.gov Identifier: NCT03385148).

## 3. Aptamer-Based Immune Strategies for TNBC Treatment

Genetic and epigenetic mutations in cancer cells lead to the presence of many tumor-associated antigens that the IS recognizes as nonself and, therefore, destroys mutated cells. However, it is well known that cancer cells evolve several mechanisms for escaping from immune destruction and changing the surrounding microenvironment in their favor, resulting in tumor growth, invasion, and metastasis [53,54,55].

**Figure 4:**
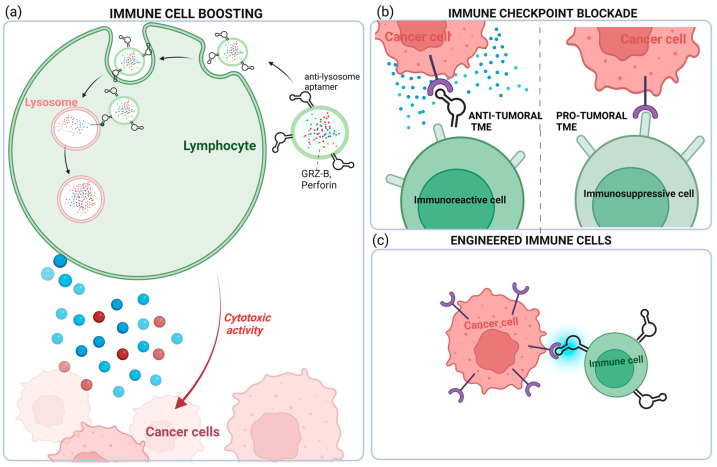
Aptamer-based strategies to restore an antitumoral immune TME. In TNBC, aptamers have been used for: (**a**) potentiating the cytotoxic activity of CD8+ T cells; (**b**) blocking immune checkpoint proteins from binding with their partners; and (**c**) restoring antitumoral TME through the recruitment of aptamer-engineered immune cells (macrophages and NK cells) to the tumor (see text for details). Created with BioRender.com (accessed on 2 March 2023).

The goal of cancer immunotherapy is to enhance or restore the IS’s ability to detect and destroy cancer cells by overcoming the mechanisms by which tumors evade and suppress the immune response. Striking aptamer-based strategies have been developed in the very last few years to restore the IS toward an antitumor condition in TNBC. As discussed below, increasing evidence shows aptamer’s ability to potentiate the cytotoxic activity of immune cells, block immune checkpoints, or recruit immune cells to cancer cells (Figure 4).

### 3.1. Tumor-Infiltrating Lymphocytes

The major types of immune cells in the TNBC microenvironment are TILs, and their presence is significantly associated with better survival outcomes in patients with early-stage untreated tumors [56]. TILs include all CD3+ T cells, which may promote tumor destruction (CD8+ cytotoxic T cells) and an antitumor response (CD4+ T-helper 1) or limit antitumor immune responses (CD4+ T-helper 2, including Forkhead box P3 (FOXP3) CD4+ regulatory T cells) [57,58].

Recently, Zhao et al. proposed an original strategy that exploits the targeting capability of aptamers to construct a “super-cytotoxic T lymphocyte” for enhanced antitumor response in cancer immunotherapy [59]. They generated acid-degradable metal–organic-based and lysosome-targeting nanoparticles that were loaded with perforin and granzyme B, two antitumor toxins contained in lysosomes of CD8+ T cells, and functionalized with an aptamer targeting the CD63 receptor on lysosome. Ca^2+^ was deposited on the nanoplatform to improve its biocompatibility and stability and potentiate toxin activity. The authors succeeded in using such an aptamer-guided platform (named LYS-NPs) for enriching lysosomes’ cytotoxic content of CD8+ T cells. When tested in the TNBC 4T1 mouse model, T cells preactivated with processed 4T1-specific antigens and recombined by LYS-NPs and released the lysosomal content into immunological synapses, triggering a strong antitumor reaction (Figure 4). The proposed aptamer-based immunotherapy has great potential to overcome significant challenges in T cell immunotherapy for solid tumors mainly represented by strong immunosuppressive signals, which induce low T cell activation and decreased synthesis and release of cytotoxic proteins [60].

### 3.2. Immune Checkpoint-Expressing Cells

Alatrash’s group reported that the expression of the PD-L1 gene in TNBC patients is significantly higher than in non-TNBC [19]. PD-L1, one of the major tumor cell-associated immune checkpoints, is expressed in a variety of immune cells, such as macrophages, some activated T cells, B cells, and in many solid tumor cells, including BC cells. Its receptor, the transmembrane protein PD-1, is expressed on the membrane surface of TILs, NK cells, macrophages, dendritic cells, and monocytes [61]. Binding between PD-L1 and PD-1 causes the inhibition of CD8+ TILs, transforming them into an anergic form and, consequently, cancer immune evasion.

Moreover, the PD-1/PD-L1 axis modulates within tumor cells various proliferative and survival signaling pathways such as PI3K/AKT, MAPK, JAK/STAT [62], and, very importantly, in TNBC, the activation of this axis promotes epithelial–mesenchymal transition (EMT), a phenotype associated with highly aggressive and metastatic tumors [63].

**Figure 5:**
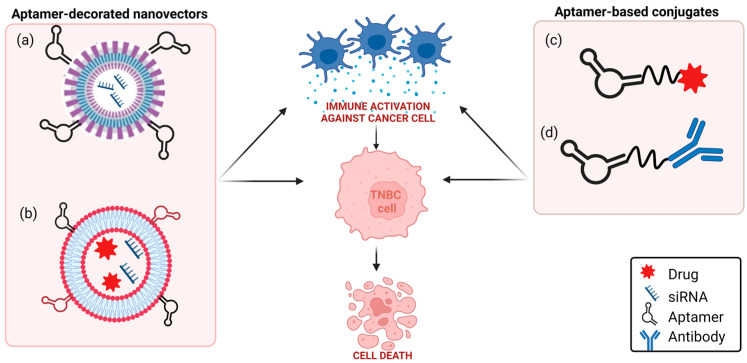
Schematic representation of aptamer-based strategies to block PD-1/PD-L1 axis in TNBC. (**a**) TNBC aptamer-decorated nanoparticles loaded with anti-PD-L1 siRNA; (**b**) anti-CD44 and anti-PD-L1 aptamer-decorated liposomes loaded with both doxorubicin and anti-IDO1 siRNA; (**c**) anti-PD-L1 aptamer conjugated to paclitaxel; (**d**) anti-EGFR aptamer covalently linked to anti-PD-L1 or anti-CTLA-4 mAbs (see text for details). Created with BioRender.com (accessed on 2 March 2023).

Different aptamer-based approaches in TNBC are currently being explored to revert PD-1/PD-L1 effects (Figure 5).

In this context, our group investigated, for the first time, a combination between an anti-PD-L1 mAb with an anti-platelet-derived growth factor receptor β (PDGFRβ) aptamer, named Gint4.T, in TNBC [64]. Gint4.T is a nuclease-resistant 2′-fluoropyrimidines (2′F-Py) RNA aptamer which binds to and inhibits PDGFRβ expressed on the surface of different human cancer cells, including TNBC cells [65], and TNBC TME components, including mesenchymal stem cells [66], and T cells [64]. Interestingly, when intravenously injected in TNBC 4T1 syngeneic mice, the aptamer strongly potentiates the effect of anti-PD-L1 mAbs in inhibiting tumor growth and lung metastases formation by acting on both tumor cells and TME components [64]. Furthermore, the combined blockade of PDGFRβ and PD-L1 causes the depletion of FOXP3+ Treg cells and an increase in CD8+ T cells and granzyme B more consistently than single monotherapies. These results lay the foundation to construct a bispecific immunoconjugate consisting of an anti-PD-L1 antibody covalently linked to Gint4.T aptamer, thus optimizing the effectiveness of combination therapy. Bispecific constructs obtained by covalently linking an anti-epidermal growth factor receptor (EGFR) 2′F-Py RNA aptamer to immunomodulators anti-PD-L1 (10_12) [67] or anti-CTLA-4 (ipilimumab) [68] mAbs were generated by Passariello et al. and proved to maintain the biological functions of both parental moieties, thus exerting a potent cytotoxic activity against BC cells.

An alternative strategy to anti-PD-L1 mAbs for PD-L1 targeting is represented by the suppression of PD-L1 through gene silencing, which has the potential to overcome some recurring obstacles of mAbs-based treatments, such as their time- and cost-consuming production, the potential for immunogenicity, and low stability. Furthermore, this strategy allows to block the intrinsic pro-tumorigenic role of cytoplasmic PD-L1 [69] that, instead, is not accessible by antibodies. The possibility of synthesizing cancer-cell-targeting aptamers with functional groups at their extremity, allowing the conjugation to nanovectors, is a striking approach to deliver, specifically to the tumor, small-interfering RNA (siRNA) cargos, loaded in the nanovector, thus overcoming the vulnerability of siRNAs to nucleases and their inability to enter into target cells. Recently, poly(lactic-co-glycolic)-block-PEG (PLGA-b-PEG)-based nanoparticles have been loaded with anti-PD-L1 siRNA and decorated with a 2′F-Py RNA aptamer able to bind and internalize specifically into TNBC cells [70,71]. The resulting aptamer-conjugated nanovectors, upon 90 min incubation on TNBC cells, efficiently delivered siRNA into target cells, which was competent to cause an almost complete suppression of PD-L1 expression [72]. Notably, aptamer-decorated nanocarriers offer the possibility to link different ligands to the surface of the NPs, thus increasing the specificity of targeting, and to encapsulate in the NPs multiple therapeutics, thus allowing for efficacious combined therapies. For example, the concomitant administration of cisplatin [40] and siPD-L1 [72] by the PLGA polymeric nanoparticles, which we equipped with TNBC aptamers, may not only promote a reduction in toxic side effects but also counteract the reported negative effect of cisplatin administration on the enrichment of PD-L1+ immune evasive TNBC cells [73]. In this regard, Kim et al. prepared a multifunctional nanosystem having two DNA aptamers conjugated on the external surface of liposomes and two different therapeutics inside nanovectors for synergistic chemoimmunotherapy in TNBC [74]. Specifically, they used, for TNBC cells targeting, the previously selected anti-CD44 [75] and anti-PD-L1 [76] DNA aptamers, each thiol-modified and covalently conjugated to maleimide groups of PEGylated-DSPE micelles by thiol–maleimide chemistry. Nanosized liposomes were loaded with both doxorubicin and siRNA interfering with the expression of IDO1, a protein that favors an immunosuppressive TME and is upregulated by doxorubicin treatment. When intravenously injected into TNBC 4T1 tumor-xenograft mice, the nanovectors strongly reduced tumor growth and inhibited metastasis formation by synergistically combining cancer-cell-targeted immunogenic cell death induction and reversal of immunosuppression [74].

Recently, different PD-L1 aptamers have been generated and tested as stand-alone antagonists, bispecific conjugates, and delivery agents of therapeutics in lung, liver, and colon tumor mouse models, which, similar to anti-PD-L1 antibodies, interfere with the PD-1/PD-L1 axis by blocking the PD-L1 (Table 2). One aptamer, named XQ-P3, has been generated by positive selection on PD-L1 overexpressing MDA-MB-231 cells by using PD-L1 knockout cells for counterselection [77]. Even if not yet tested in vivo, it appears highly effective in co-cultures of TNBC MDA-MB-231 cells and immune Jurkat cells by blocking the interaction with PD-1 and restoring T cell function. Furthermore, an XP-Q3 aptamer-paclitaxel conjugate showed anti-proliferation efficacy in PD-L1 overexpressed TNBC cells [77].

Some aptamers against PD-1 receptor have also been selected and tested in mouse models of human cancers but not yet in TNBC (reviewed in [85]).

### 3.3. Macrophages

Tumor-associated macrophages (TAMs) are among the most abundant immune cells in the TME of a broad range of cancers and may act to promote or suppress antitumor immune responses [86,87]. Indeed, due to their high degree of plasticity, they shift to two diverse phenotypes in response to various micro-environmental stimuli: classically activated, pro-inflammatory M1, and alternatively activated anti-inflammatory M2, which display a differential expression profile to cell surface markers and different cytokine and chemokine production. M1 macrophages typically exert antitumor functions, while M2 macrophages promote tumor progression. In most aggressive tumors, including TNBC, TAMs tend to resemble an M2-like phenotype that largely accounts for the failure of conventional therapies and immune checkpoint inhibition therapies. For this reason, several innovative immunotherapeutic approaches aim to target and deplete M2 macrophages or reprogram them to the desired phenotype [88,89].

In order to select aptamers targeting human M2-like macrophages, the first cell-SELEX approach was applied to human macrophages derived from monocytes of several donors and polarized to the M2-like phenotype [90]. Although the best M2-targeting DNA aptamer coming from the selection was not able to discriminate the target cells from undifferentiated M0-like and monocytes and also bound at a lower extent to M1-like macrophages, it rapidly internalized into CD14+ monocytes, thus holding potential for monocyte-targeted drug delivery applications.

Another striking application of aptamers for solid tumor immunotherapy consists of potentiating M1 macrophage specificity for tumor cells by engineering them with cancer-cell-targeting aptamers. Chimeric antigen receptor T (CAR-T) cell immunotherapy, which infuses patients with CAR-T cells, has shown great efficacy in the treatment of some leukemias and lymphomas but only modest results in solid tumors due to the difficulty of penetrating tumors [91]. Because of the intrinsic capacity of macrophages to penetrate tumor tissues, several approaches have been recently proposed that genetically engineer them to express chimeric CARs (CAR-M) for targeting tumor cells and initiate a targeted antitumor response [92]. In order to overcome major drawbacks associated with traditional CAR-M therapies, such as the low reproducibility of engineered proteins and safety issues, Qian et al. proposed a new CAR-M approach based on the use of aptamers [93]. The murine stable macrophage cell line, RAW 264.7, was first incubated with an azide-containing metabolic glycoprotein labeling reagent and lipopolysaccharide to generate azido sugars on the M1 cell surface. Then, M1 cells were conjugated by click chemistry reaction to both the AS1411 aptamer, which binds to nucleolin expressed on several cancer cells, and a PD-L1 aptamer, for simultaneous tumor targeting and immune checkpoint blockade. Importantly, in vivo imaging of mice bearing 4T1 TNBC and intravenously injected with M1 cells, functionalized with fluorescent aptamers, showed a greater accumulation in tumors compared with unmodified M1 cells. Furthermore, when tested for antitumor activity, the dual-aptamer-engineered M1 caused a strong reduction in tumor growth and metastasis formation, which was accompanied by immune TME reprogramming with increased T cell infiltration in the tumor and enhanced T cell cytotoxicity.

**Figure 6:**
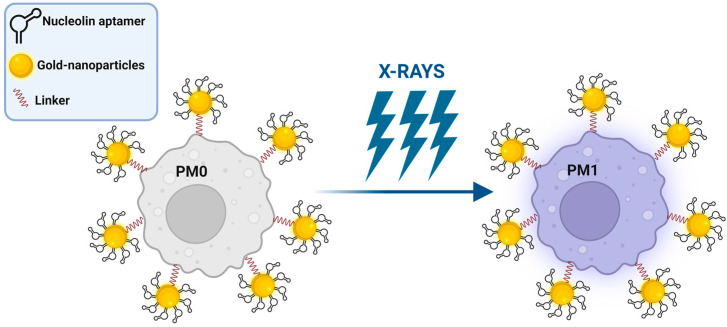
Schematic representation of PSA macrophage engineering strategy (see text for details). Created with BioRender.com (accessed on 2 March 2023).

Alternatively, Chen et al. proposed polyvalent spherical aptamers (PSAs) as a macrophage engineering strategy [94]. PSAs were generated through the functionalization of gold nanoparticles with both the thiol-modified AS1411 aptamer and a DNA linker that carries, at the free extremity, a functional group for reacting with azide tags created on M0 macrophages through the abovementioned metabolic labeling and biorthogonal click reactions (Figure 6). The phenotypic transformation of engineered non-polarized macrophages into the M1 subtype was activated by X-rays in vitro and confirmed in mice bearing 4T1 tumor xenografts, causing potent tumor-specific killing without signs of systemic toxicity.

These studies clearly show how the integration of aptamers in macrophage-guided immunotherapy is an effective strategy to enhance the antitumor effect of the IS.

### 3.4. Natural Killer Cells

NK cells are cytotoxic lymphocytes belonging to the innate IS, able to produce inflammatory cytokines and chemokines. They are called the “first line of defense” because, different from T lymphocytes, they do not express antigen-specific T cell receptors but act against mutated cells without prior sensitization or clonal expansion [95]. NK cell adoptive immunotherapy failed to show efficacy in the treatment of solid tumors, partly due to the immunosuppressive TME and lack of NK cell specificity to the tumor [96].

Therefore, among the approaches for improving NK cell anticancer therapeutic efficacy, a great effort is focused on conferring cancer specificity through the expression of CARs or the conjugation of tumor-targeting ligands [97]. Zu and colleagues explored aptamers as active cancer-targeting agents by linking an aptamer, capable of specifically recognizing the CD30 receptor, on lymphoma cells to the surface of either an NK commercial cell line or NK cells obtained from three healthy donors [98]. This DNA-type aptamer was previously selected by the same group through a hybrid SELEX approach, in which steps of selection on CD30+ lymphoma cells were followed by selection steps on the CD30 recombinant protein [99]. The aptamer was modified at the 3′ end with lipophilic double C18 hydrocarbon chains for anchoring into the membrane of NK cells, which are so guided specifically to lymphoma cells to kill them [98]. More recently, the same authors applied the same approach in TNBC by attaching a DNA aptamer capable of binding a not-yet-known protein expressed on TNBC cells to the surface of NK cells. Aptamer-engineered NK cells inhibited lung metastasis from MDA-MB-231 cells intravenously injected in mice without side toxicity to normal tissues [100].

In order to further enhance the tumor-specificity of NK cells in solid tumors, dual aptamer-equipped NK cells were generated by using both an aptamer targeting hepatocellular carcinoma cells and the AptPD-L1 aptamer [81]. The resulting engineered NK cells were more effective than cells unconjugated or conjugated with only one of the two aptamers in inhibiting the growth of hepatocellular carcinoma in adoptively transferred mice. Another limit to the efficiency of immunotherapy with NK cells is their insufficient infiltration in solid tumors. Once again, aptamers have proved to be excellent tools for overcoming this problem. Hock’s group generated a bispecific aptamer-based conjugate capable of simultaneously binding to c-Met, a receptor highly expressed on several tumor cells, and to the Fcg receptor III (CD16a), a protein expressed on NK cells [101]. The conjugate is made up of the two highly specific c-Met and CD16a DNA aptamers that were fused by different linkers, preserving the ∼65 Å-ideal distance for simultaneously binding to the two receptors. The conjugate was able to efficiently mimic antibody-dependent cellular cytotoxicity by recruiting NK cells to cancer cells. Later, the same CD16 aptamer was fused to a PD-L1 DNA aptamer to generate a construct able to both recruit NK cells to PD-L1+ tumor cells and impair the PD-1/PD-L1 immunosuppressive axis by reactivating TILs against tumor cells in tumor-bearing mice [82]. This approach is particularly indicated for those solid tumors with high levels of PD-L1, such as TNBC.

## 4. Conclusions

The recent studies discussed here clearly demonstrate the great potential of oligonucleotide aptamers to amplify our IS to fight cancers. Aptamers can be used as anticancer agents in the same way as mAbs but are cheaper, produced more rapidly and at a greater reproducibility, and less immunogenic than antibodies. However, it must be recognized that the arrival of aptamers in the clinic is proving to be slower than expected; in fact, although more than 30 years have passed since the first SELEX [25,26], only three aptamers are currently in clinical trials for cancer treatment [51].

This slowdown is mostly due to some challenges that limit aptamers’ efficacy in patients, such as their uncertain stability and half-life, especially in the complex and continuously evolving microenvironment that surrounds the tumor. Nevertheless, the astonishing strategies that have been developed in the very few last years to overcome the abovementioned limiting issues and the recent progress in aptamer discovery and modifications for adapting them to any desired applications make it reasonable to argue that the practice use of aptamers will soon be realized for cancers such as TNBC, which urgently need new therapeutic options.

## Figures and Tables

**Table 1:** Comparative aspects between the aptamers and antibodies for cancer therapy.

	Aptamer	Antibody
Molecular feature		
− Composition	Nucleotides	Amino acids
− Molecular weight	5–15 kDa	150–180 kDa
− Resistance to harsh conditions(i.e., pH, temperature)	High	Low
Manufacturing		
− Production	Easy and cheap	Labor-intensive and expensive
− Batch-to-batch variation	None	Significant
− Versatility tochemical modifications	High	Limited
− Discovery time	~2–8 weeks	~6 months
Target recognition		
− Binding affinity	High (Kd, pM-nM)	High (Kd, pM-nM)
− Minimal target size	~60 Da	~600 Da
− Target activity modulation	Yes	Yes
In vivo behavior		
− Nuclease resistance	Sensitive (chemically unmodified)	Resistant
− Tissue penetration	High	Low
− Renal excretion	Rapid (chemically unmodified)	Slow
− Immunogenicity	Low or absent	High

**Table 2:** Summary of anti-PD-L1 aptamers as cancer therapeutics.

Aptamer	SELEX Approach	Target (Positive Selection)	Composition	Application	Ref.
AptPD-L1	Nitrocellulose filter SELEX	Recombinant human PD-L1	DNA	Inhibition of tumor growth by T cell function restoration and TME modification in colorectal-cancer- and lung-cancer-bearing mice	[76]
				Tetravalent aptamer-Holliday Junction nanostructure for improving the efficacy of monovalent AptPD-L1 in PD-1/PD-L1 blockade in colon-cancer-bearing mice	[78]
				Bispecific anti-CTLA-4/PD-L1 aptamer for dual immune checkpoints blockade in hepatocellular-carcinoma-bearing mice	[79]
				AptPD-L1-functionalized and oxaliplatin-loaded metal–organic framework nanoparticles for combined chemotherapy, PDT, and immune checkpoint blockade in colon-cancer-bearing mice	[80]
				Nanoliposomes functionalized with AptPD-L1 and anti-CD44 aptamers and loaded with doxorubicin and IDO1 siRNA for synergistic chemoimmunotherapy in TNBC-bearing mice	[74]
				NK cells recruitment to PD-L1+ tumor cells	[81,82]
XQ-P3	Cell-SELEX	PD-L1 overexpressing MDA-MB-231 cells	DNA	PD-1/PD-L1 blockade in cocultures of MDA-MB-231 cells and immune cells Delivery of Paclitaxel to PD-L1 overexpressed TNBC cells	[77]
N5, S42	Capillary electrophoresis SELEX	Recombinant human PD-L1	Threose nucleic acid	Blocking the interaction of recombinant PD-L1 with PD-1-expressing cells (N5, S42) Inhibition of tumor growth in colon-cancer-bearing mice (N5)	[83]
PL1	Cell-SELEX	PD-L1 overexpressing CHO-K1 cells	Phosphorothioate DNA	PD-1/PD-L1 blockade and inhibition of tumor growth in colon-cancer-bearing mice	[84]

## Data Availability

Not applicable.
